# The complete mitochondrial genome of *Allantus togatus* (Panzer, 1801), in view of possible cryptic species

**DOI:** 10.1080/23802359.2021.1899875

**Published:** 2021-03-24

**Authors:** Jiyun Yang, Zemin Sun, Meicai Wei, Gengyun Niu

**Affiliations:** College of Life Sciences, Jiangxi Normal University, Nanchang, China

**Keywords:** Cryptic species DNA barcoding, *Allantus*, mitochondrial genome, Allantinae

## Abstract

The complete *Allantus togatus* (Panzer, 1801) mitogenome was determined and analyzed. The mitogenome contains typical 37 genes with identical order to *Allantoides luctifer* mitogenomes. Phylogenetic analysis revealed that *A. togatus* clustered together with *A. viennensis*. The wide genetic distances found between lineages of *A. togatus* lead to the assumption of cryptic species. These complete mitogenomes provide valuable information at the genomic level that can be utilized to sustain bioresources to deepen the understanding of cryptic diversity within Allantinae.

*Allantus togatus* (Panzer, 1801), a widely distributed sawfly in Europe, belongs to the Allantinae of Tenthredinidae. As this species is the type species of genus *Allantus* Panzer, 1801, its phylogenetic status is very important for understanding the phylogeny of *Allantus* and its relatives, as well as the evolution of the cryptic lineages of *Allantus* species. Furthermore, the potential occurrence of cryptic species is mentioned due to their individual variability (Schmidt et al. 2017) in this genus. Species with a wide distribution require a broad sampling and population and biogeography studies to determine the reliability of the species. Five barcodes of *A. togatus* from the type locality, Germany, have been published (Schmidt et al. 2017). Herein, we reported the complete *A. togatus* mitogenome from Spain.

Genomic DNA from one female *A. togatus* individual collected from Spain (Valencia, N39°41′45.6″, W0°25′19.2″) was used for genome sequencing. The specimen (CSCS-Hym-MC0142) was deposited at the Asia Sawfly Museum, Nanchang (ASMN) (Meicai Wei, weimc@126.com). Paired-end reads were sequenced using Illumina Hiseq 4000 platform at NOVO gene Ltd. (Tianjin, China). Approximately 11 Gb of paired-end (150 bp) sequence data was yield. A total of 38,288,919 reads were *de novo* assembled using MitoZ (Meng et al. 2019).

We then assembled a consensus scaffold of the complete mitogenome using the reference-based assembly approach in Geneious (Biomatters Ltd., Auckland, New Zealand). *Allantoides luctifer* (KJ713152) was used as the seed sequence, with the mean depth of coverage being 10864. Annotations of tRNAs were generated in MITOS web server (Bernt et al. 2013). Protein-coding genes (PCGs) were annotated based on the related reference genomes. Phylogenetic analysis was conducted by IQtree (Trifinopoulos et al. 2016) based on COI genes (Alignment length: 1569 bp) of *Allantus* using *Asiemphutus rufocephalus* and *Allantoides luctifer* as outgroups.

The complete *A. togatus* (MW464859) mitogenome comprises a circular DNA molecule measuring 15,480 bp in length. The mitogenome contains typical 37 genes and a control region of 516 bp in length. The overall A + T content of the complete mitogenome is 80.7%. All the PCGs were initiated with ATN (ATT, ATA, and ATG) codon. Among those genes, four PCGs (*atp8*, *nad2*, *nad5*, *nad6*) initiated from ATA, sever PCGs (*atp6*, *cox1*, *cox2*, *cox3*, *cob*, *nad4*, *nad4l*) initiated from ATG and two PCGs (*nad1*, *nad3*) initiated from ATT. Ten PCGs (*atp6*, *atp8*, *cob*, *cox1, cox2*, *cox3*, *nad1*, *nad2*, *nad4l*, *nad6*) have a TAA termination codon, except *nad3* was finished with TAG, *nad5* was finished with TAC, and an incomplete terminal codon T was found in *nad4*. Compared with the ancestral insect mitochondrial genome (Boore 1999), *trnI* was located upstream of *nad2*, *trnQ* (–)-*trnM* (+) clusters were rearranged to *trnM* (–)-*trnQ* (+). It maintained the same gene order as previously reported in Allantinae. There was three gene overlapping regions that appeared among *atp6*-*atp8* (7 bp), *atp6*-*cox3* (1 bp), *nad6*-*cob* (1 bp). There were nine intergenic spacers with a total length of 137 bp in 18 locations and varied in size from 1 to 27 bp, with the longest located between *nad1* and *trnS1*.

The placement of *A. togatus* in the unsaturated PCGs tree was consistent with the traditional view. Maximum likelihood inference based on COI showed that specimens identified as *A. togatus* formed one clade, which was divided into two sub-clades (Figure 1). One containing five sequences from Germany (Mean *p*-distance within the German population: 1.6%) was very distantly related (Mean *p*-distance 6.9%) to the other Spanish clade. The sister group *A. viennensis*, in the same situation, can be divided into three sub-clades, each containing specimens from Belgium, Iran, and Germany, respectively (Average mean *p*-distance between groups: 4%). The COI sequences of six *A. togatus* have 373 identical sites (91.6% pairwise identity). The difference between the Spanish individual and the German population includes 23 substitutions. Among them, one substitution results in a change to the amino acid.

**Figure 1.**
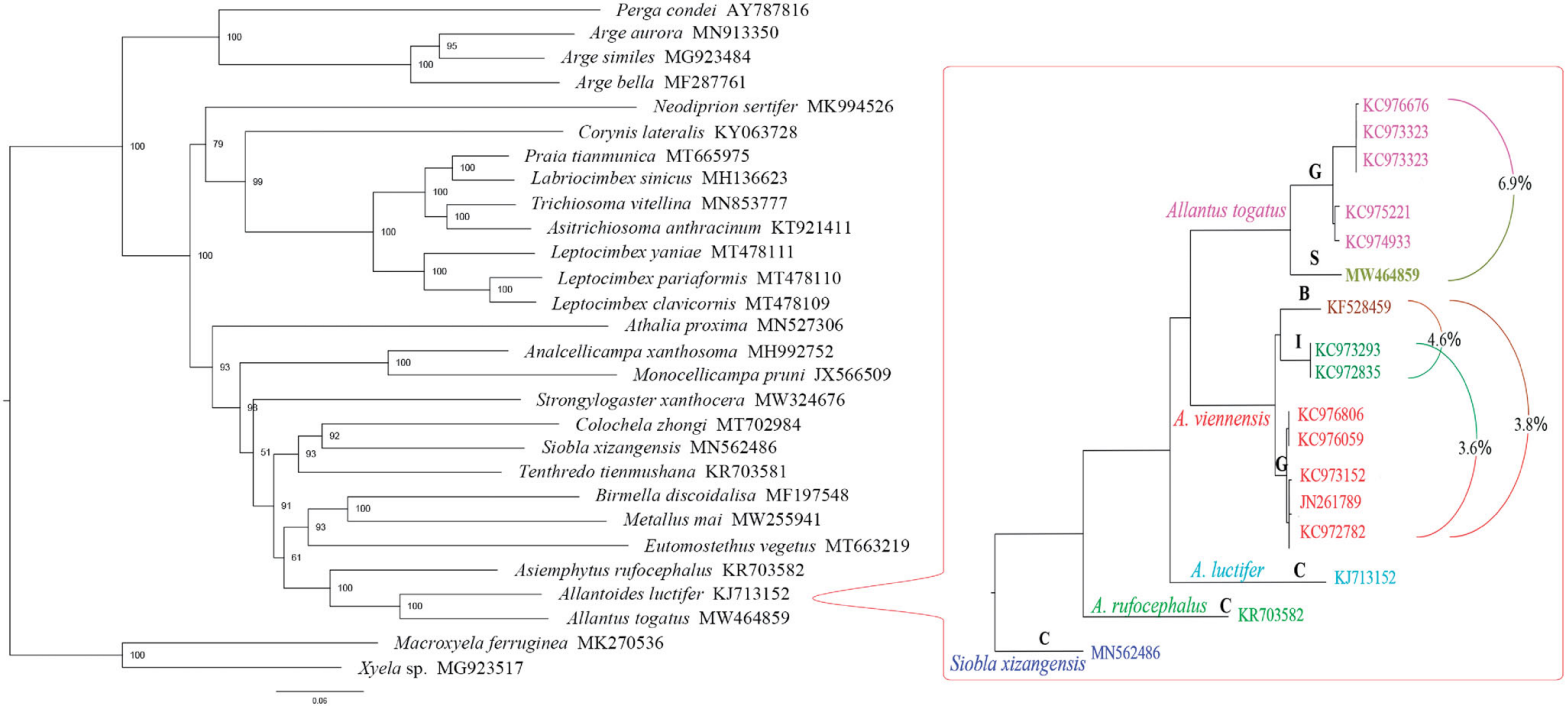
Maximum likelihood trees based on 26 Tenthredinoidea sequences from the nine unsaturated PCGs (left) and on the alignment (1569 bp) for 14 COI sequences of *Allantus* species (right). P-distances between clades are provided, to highlight the significant genetic differences within *A. togatus* and *A. viennensis*.

The significant genetic differences within *A. togatus*, shows that DNA barcoding might be a promising tool. But, to better understanding the fauna of European sawflies, more population and biogeography studies are needed. Thus, it is necessary to collect and compare mt-genomes across Europe, allowing a comprehensive revision of the sawfly taxa in Europe.

## Data Availability

The genome sequence data that support the findings of this study are openly available in GenBank of NCBI at [https://www.ncbi.nlm.nih.gov] (https://www.ncbi.nlm.nih.gov/) under the accession number MW464859. The associated BioProject, SRA, and BioSample numbers are PRJNA674994, SRR13065511, and SAMN16686211 respectively.
